# Perceived availability of future care and depressive symptoms among older adults in China: evidence from CHARLS

**DOI:** 10.1186/s12877-020-1435-1

**Published:** 2020-01-30

**Authors:** Merril Silverstein, Cathy Honge Gong, Hal Kendig

**Affiliations:** 1 Centre for Research on Ageing, Health and Wellbeing (CRAHW), Research School of Population Health (RSPH), Australian National University (ANU), Canberra, ACT Australia; 2 Australian Research Council (ARC) Centre of Excellence in Population Ageing Research (CEPAR), Sydney, Australia; 3 Aging Studies Institute, Syracuse University, Syracuse, NY USA

**Keywords:** Informal care, Depressive symptoms, Older Chinese, Social support theory, Control theory, Perceived care support

## Abstract

**Background:**

Major concerns have arisen about the challenges facing China in providing sufficient care to its older population in light of rapid population ageing, changing family structure, and considerable rates of internal migration. At the family level, these societal changes may produce care uncertainty which may adversely influence the psychological wellbeing of older individuals. This paper applies social support and control theories to examine the relationship between perceived availability of future care and psychological wellbeing of older adults in China, and how this relationship is moderated by economic insufficiency, health vulnerability, and urban/rural context.

**Methods:**

Analyses are based on data from the China Health and Retirement Longitudinal Study, a multi-panel nationally representative household survey of the Chinese population aged 45 years and older. Data are taken from 2013 and 2011 waves of the study, with an initial sample size around 17,000, in which around 11,000–14,000 respondents are used for our final regression model. The score of depressive symptoms was measured in both waves with the Center for Epidemiologic Studies Depression Scale (CES-D10), and perceived availability of future care was measured in 2013 by asking respondents the question “Suppose that in the future, you need help with basic daily activities like eating or dressing, do you have relatives or friends (besides your spouse/partner) who would be willing and able to help you over a long period of time (yes/no)?”

**Results:**

Multivariate regression analysis revealed that uncertainty regarding future care support was associated with greater depressive symptoms even after controlling for factors confounded with care uncertainty such as family structure, socio- economic status, and a lagged measure of depression. Further, older adults without an anticipated source of care faced double jeopardy in their depressive symptoms if they also experienced functional limitations.

**Conclusions:**

Considering rapid aging of the Chinese population, anticipated increases in chronic disease burden, and possible attenuation of filial care, this analysis suggests that older adults in China may increasingly face health and social conditions detrimental to their mental health. Polices that remedy these concerns should be discussed, developed and implemented.

## Background

Mental health problems, such as depression, are increasingly recognized as a major threat to the capacity of older adults to fully participate in daily life [1, 2]. China represents an anomalous case in this regard, with lower than expected prevalence of depression in its older population [3–5]. This deviation has been explained by cultural factors found in most East Asian nations, most notably strong filial responsibility and high levels of respect and family support for older people deriving from Confucian values of filial piety [6, 7]. However, recent studies have found the prevalence of depression among older adults in China has risen precipitously over recent decades [8], while at the same time families have grown smaller, altering the contours of family life of older individuals [9]. Urbanization and societal developments in China have arguably increased uncertainty about care availability among older adults, with commensurate risks to their mental health [10]. Consequently, it is important to explore the relationship between perceived future care support and depression in contemporary China.

In this investigation, we used data from a nationally representative data set of older adults in China to examine whether perceived availability of future care (PAFC) predicts depressive symptoms independently, as well as in conjunction with health, social, family, and economic factors. We rely on social support and control theories as explanatory frameworks for understanding how uncertainty about care may adversely influence mental health in the older population of China.

### Social support theory

We first rely on the stress buffering and double-jeopardy hypotheses within social support theory [11] which state that resources moderate the impact of unexpected and stressful events on well-being outcomes [12, 13]. We argue that economic, health, and community resources may mitigate the negative impact of lacking an expected care provider as a form of stress-buffering, while economic, health, and community deficits may magnify the negative impact of lacking an expected care provider as a form of double-jeopardy [14]. The double-jeopardy hypothesis suggests that financial insufficiency, poor functional health, and living in a rural area elevate the risk impact of care uncertainty on emotional distress by magnifying feelings of precariousness.^1^ Stress-buffering and double-jeopardy perspectives predict the same data pattern, making it difficult to adjudicate which is the preferred explanation, as each is consistent with the other in predicting an interaction effect.

### Control theory

Control theory provides a complementary vantage point from which to view the implications of anticipated care on psychological well-being from the view of a psychologist. Control theory derives from the assumption that achieving mastery over life’s challenges is an important prerequisite for reducing stress caused by adverse contingencies related to old age [11]. An important element of mastery are the resources that one can bring to bear in order to meet those challenges [15, 16]. In this context, resources include both cognitive/intrinsic traits and social/extrinsic supports, and sometimes both [17–19]. For example, the perception that social support will be available to satisfy needs caused by physical decline in later life represents the cognitive appraisal of one’s control over a potential external resource. Lacking a sense of personal control over meeting future needs has been found to have adverse consequences for health and well-being of older adults [19].

Two aspects of control theory are relevant to our investigation. First, anticipating that care needs will be met is a desired state and an important aspect of security about future uncertainties. Being able to count on others produces confidence that the social environment can be effectively managed. Second, expectations about the social environment are shaped by cultural and political contexts. China represents a case-in-point by having strong norms of filial duty and a weak social safety net for its vulnerable inhabitants [7, 20], both of which raise the stakes of expecting a weak social support system in old age. The dictates of filial piety enhance expectations for support and care from family members—adult children in particular—the violation of which may lead to a sense of loss and increased distress [7].

### Factors related to care expectation

The expectation that a care provider will be available when needed is likely to be associated with deficits (and, conversely, with resources) that are also related to depression. Examples of such deficits include poor health, financial inadequacy, lack of kin supply, social isolation, and weak public and community supports [21, 22]. Consequently, we examine the association between perceived availability of future care and depression controlling for these possible confounding factors. According to the stress buffering and double-jeopardy hypotheses, we specifically focus on economic well-being, functional health and urban/rural residency as important confounders and moderators of the association between perceived availability of future care and mental health.

Economic strain tends to be associated with psychological distress and its absence associated with psychological well-being [23, 24]. This result has been found in older Asian populations, including China [8, 21, 25] and Vietnam [26].

Health is one of the most robust correlates of late-life mental health, Studies across a variety of nations consistently find that poor health—whether indicated by chronic diseases, functional limitations, or pain—is associated with depression. Studies in the United States have found that the perceived availability of social support can buffer the impact of age-related deficits and vulnerabilities on depression in later life [15, 27, 28], a finding also demonstrated in China [8, 21, 29], Taiwan [29], Korea [25] and Vietnam [26].

Rural residency is also positively associated with depression among older adults in China [22] . This finding may be explained by the unique challenges faced by rural elders such as inadequate pension coverage [22], low availability of mental health professionals, and underdevelopment of public programs serving vulnerable elders (for a review, see [10]). In addition, rural elders may be left behind in their natal villages by migrant children [30].

### Social determinants of depression

Psychological wellbeing is an important component of later life quality [2]. The previous studies in China [8, 21, 22] and Asian countries [25, 26] found that psychological wellbeing differs significantly among older population by age, gender, marital status, social and economic status, physical health and social support. In more details, (1) education, financial status (material hardship, expenditure, or age subsidy), and chronic diseases were significant and important predictors for depression in China, Vietnam and Korea; (2) females and older people with ADLS and pains had a significant higher depression score in China and Vietnam; (3) Emotional support could prevent both the onset and progression of depression among older adults in Viet [26]; (4) multiple factors, including age/cohort, functional problem, marital status, urban/rural residence, lack of contacts with children, perceived future care support, active participation, senior center, community amenities, childhood health were all associated with depression in China [8, 21, 22].

Literature also documents the mental health benefits of perceiving that a support provider will be available in later life and perceived availability of support-even more than actual support itself-offers comfort and security to older adults, with positive consequences for their emotional well-being [31–34]. A study of the older population of Taiwan found that perceived availability of a support provider was protective of mental health [35].

Similarly, perceived future care support was shown to be negatively associated with depression among older adults based on a pilot survey data in two Chinese provinces in 2008 [21]. Nevertheless, the perceived availability of informal care has rarely been studied nationally in China and how functional health, financial adequacy, and urban/rural residency will moderate the relationship between expected care support and depressive symptoms has not been fully investigated.

In this study, we are filling the research gap by, (1) using data from a nationally representative data set of older adults in China; (2) applying social support and control theories as explanatory frameworks for understanding how uncertainty about care may adversely influence mental health in the older population of China; (3) investigating how functional health, financial adequacy, and urban/rural residency will moderate the relationship between expected care and depressive symptoms.

### Hypotheses

In this investigation, we rely on the social support and control theories as the frameworks to hypothesize that (1) older adults in China who do not expect to have an available care provider will have more depressive symptoms than those who have such an expectation (to estimate the initial relationship between depression and perceived future care support). Further, we hypothesize that (2) this relationship will be at least partially explained or confounded by the presence or absence of health, financial, and social deficits (to estimate the true relationship between depression and perceived future care support after controlling for other variables). Finally, we rely on stress-buffering and double-jeopardy paradigms to hypothesize that (3) functional health, financial adequacy, and urban/rural residency will moderate the relationship between expected care and depressive symptoms either because: (a) resources ameliorate the negative impact of having no expected care provider (stress-buffering) or (b) deficits elevate the negative impact of having no expected care provider (double-jeopardy) (to estimate the moderating effect of rural-urban residents, financial conditions, and health).

## Methods

### Sample

Data used for our analysis derived from the China Health and Retirement Longitudinal Study (CHARLS), a nationally representative survey of the population 45 years or above living in China. Beginning in 2011 and continuing biannually, CHARLS collects information on a variety of health, social, family, and financial characteristics through face-to-face interviews in respondents’ homes [36]. For our analysis, we used the 2013 wave of data which totaled 18,246 respondents, of whom 14,988 also participated in the previous 2011 wave from which the lagged depression measure derived. Sample weights were applied in our analyses to account for design effects and survey non-response*.*

### Measures

The dependent variable of interest, the score of depressive symptoms, was measured with 10 questions from the Center for Epidemiologic Studies Depression Scale (CES-D10) [8, 22, 25]. Respondents were asked how frequently in the last week they: were bothered by things; had trouble concentrating on things; felt depressed; felt everything was an effort; felt hopeful about future; felt fearful; had restless sleep; was happy; felt lonely; and could not get going. After reverse scoring the two positively worded items, we assigned a score from 0 to 3 for each item as follows: 0 for 0 days, 1 for 1–2 days; 2 for 3–4 days; 3 for 5–7 days. Scores of these 10 items were summed to create an additive scale score ranging from 0 to 30, with higher scores indicating more depressive symptoms. The reliability of depression items was tested using Cronbach’s alpha and found to be satisfactory at each wave (alpha = 0.76 in 2013 and alpha = 0.81 in 2011).

The key independent variable, perceived availability of future care (PAFC), was measured by the following question “Suppose that in the future, you needed help with basic daily activities like eating or dressing, do you have relatives or friends (besides your spouse/partner) who would be willing and able to help you over a long period of time? The response option was *yes* or *no*. Coding this variable in such a manner allows us to discuss *unavailability* of support as a risk factor for depression. Additionally, for respondents answering “yes”, a follow-up question was asked about whether the source of expected care support would be children, other relatives, or friends. Examining those with care expectations by source of care (not shown) revealed the importance of off-spring in care availability: 98% of respondents expected care from their children while only 2% expected care from other relatives or friends. This provides evidence to support the existing of traditional filial piety culture in China and the importance to control for the number of children, the frequency of contacts/visits and living arrangement between parents and children into the final regression models.

We chose potential control variables based on previous studies of the determinants of depression in China and other Asian countries [21, 22, 25, 26, 29, 37–39]. These variables were categorized as demographical, socio-economic/finance, health and social factors. Demographics included age and gender. Age was divided into four groups: 45–54, 55–64, 65–74 and 75+. Socio-economic status was represented measured by the highest educational level achieved, perceived living standard, and urban/rural residency. Education was measured as the highest level of education achieved based on three categories: Primary schooling or less; secondary schooling; and college or higher degree. Relative living standard was assessed by responses to the question “Compared to the average living standard of people in your city or county, how would you rate your standard of living?”^2^ Response options were *much better, a little better, about the same, a little worse, much worse*, collapsed into three categories corresponding to better, same, and worse [40]. Worse than the average relative living standard is treated as having financial strain/uncertainty.

Urban and rural residency was determined by the most recently published statistical standard by the Chinese National Bureau of Statistics based on an area’s social and economic development [40].

Health factors included limitations in activities of daily living (ADL), limitations in instrumental activities of daily living (IADL), number of chronic diseases, functional loss, poor memory, level of chronic pain, and childhood health. ADL limitation was indicated if the respondent -reported difficulty performing any of the following basic activities: bathing/showering, eating, dressing, getting into or out of bed, using toilet, or controlling urination and defecation. IADL limitation was indicated if the respondent reported difficulty in any of the following household activities: doing household chores, preparing hot meals, shopping for groceries, managing money, and taking medications. Chronic diseases were assessed as the number of diagnosed health conditions categorized as none, one, two to three, and more than three. Functional loss was indicated by whether respondents reported any of the following disabilities: brain damage/mental retardation, vision problem (blind or half blind), hearing problem (deaf or half deaf) and speech impediment (full or half).” Self-reported memory was assessed with the question: “How would you rate your memory at the present time?”. A dichotomous variable was created differentiating excellent/very good/good (=0) from fair/poor(=1). Level of pain was ascertained by the question “Yesterday, did you feel any pain?”, of pain was assessed as no pain (1), a little pain (2), some pain (3), quite a bit of pain (4), and a lot of pain (5). Childhood health was measured by asking “How would you evaluate your health during childhood, up to and including age 15: excellent (1), very good (2), good (3), fair (4), poor (5). Both level of pain and childhood health were continuous variables controlled in the multivariate regression models.

Social factors included family structure, intergenerational arrangements (contact/co-residence/geographic distance), and social activities. Family structure was measured by marital status and number of children. Marital status was operationalized as currently married or cohabitating, formally married (divorced, separated or widowed), and single or never married. Number of living children was categorized into four groups: no children, one child, two children, and three or more children. Intergenerational contact was measured as the most frequent form of contact (face-to-face visits, phone, email and Internet) with non-coresident adult children. Contact frequency was categorically assessed as daily, weekly, monthly, once per year, or less than once per year. Geographic distance from children was based on the location of the closest child: same/adjacent household/dwelling/courtyard, another household in your village/neighborhood, another village/neighborhood in your city/county/district, or outside your city/county/district. Three categories were generated from these responses: Having at least one child living in the same or adjacent household, having a child living in the same village/neighborhood but not co-residing or living adjacent, and having all children living beyond the village/neighborhood. Participation in social activities was assessed by whether or not respondents participated in each of three types of activities in the last month: *leisure activities* (e.g. interacting with friends; playing Ma-jong or cards, participating in a club or community-related organization); *helping activities* (e.g. providing help or care for family, friends, neighbors or others who do not live with you and did not pay you); and *educational* activities (e.g. attending an educational or training course for stock investment; using the Internet).

The CHARLS interviewed both the husband and the wife in a same household as long as they were both aged over 45, as one of them being the “main respondent”. We note that the CHARLS study randomly selected one respondent per household (58% are main respondents and 41% are the spouses of the main respondents) to report family-level information, including number and location of children, as well as contact and exchanges of money and support with children. This method resulted in about half of our sample having no directly reported data about children. We empirically handled this issue by generating a category for “non-response” in order to retain these cases in our analysis. As a robustness check, we also estimated models borrowing values from reporting respondents and found very similar results (not reported).

### Analytic approach

Previous studies on risk factors of depressive symptoms among older adults in China generally find that depression correlates with being older, female, retired, physically disabled, chronically ill, financially stressed, and low educated, and having weak social and family support systems [6, 8, 21, 22, 41]. We account for these factors in our predictive models of depressive symptoms as a function of expected future care.

Emotional distress deriving from uncertainty in meeting future care needs may be assuaged by financial resources (allowing the purchase of private services), good functional health (rendering its impact less consequential), and living in an urban area (where a relatively strong service infrastructure and relatively weak filial norms shift the burden away from families).

In order to select the most relevant control variables, we used univariate OLS regressions predicting depressive symptoms to identify plausible variables with coefficients significant at <.10 and r-squares higher than 0.01. Variables that did not meet these criteria were excluded from our analysis. Selected variables are presented in Table 1. Multivariate OLS (Ordinary Least Squares) regression was then used to explore the association between perceived future care availability and depressive symptoms, first with only demographical variables controlled, then sequentially adding health, socioeconomic, and social variables until all control variables were entered to the final model. We used this hierarchical estimation approach to assess the unique contribution of each variable grouping toward explaining the association between perceived future care and depressive symptoms.

**Table 1:** Perceived availability of future care and mean depression scores by sample characteristics

Sample characteristics	Sample size (n)	Population distribution (%)	Perceived future care availability (%)	Depression score (mean, 0–30)
Perceived availability of future care				
Expected future care support (Yes)	11,886	70.7	100.0	7.1
Expected future care support (No)	5010	29.3	0.0	9.1 *
Predisposing factors				
Aged 45–54 (Ref.)	5920	33.4	71.5	7.3
Aged 55–64	6694	35.2	69.0 *	8.0*
Aged 65–74	3736	19.7	70.1	8.2*
Aged 75+	1855	11.7	74.6*	7.8*
Male (Ref.)	8806	48.4	69.1	6.9
Female	9429	51.6	72.3*	8.6*
Financial factors				
Rural (Ref.)	10,881	58.7	71.6	8.4
Urban	7361	41.3	69.4*	6.8*
Under primary (Ref.)	8182	44.9	71.5	8.9
School without degree	9633	52.9	70.4	7.0*
College and above degree	414	2.2	63.5*	5.2*
Better living standard (Ref.)	503	3.6	69.7	6.0
About same living standard	3595	28.1	72.3	6.4
Worse living standard	8891	68.4	69.0	8.5*
Living standard not reported	5634	~	72.6	7.5
Health factors				
With ADLs (Yes)	1114	9.4	69.0	12.3*
With ADLs (No) (Ref.)	11,360	90.6	69.3	8.8
ADLs not reported	5621	~	73.9	5.2
With IADLs (Yes)	2628	14.8	70.2	11.4*
With IADLs (No) (Ref.)	15,402	85.2	70.8	7.3
No disease (Ref.)	4846	33.7	72.8	6.5
One disease	4430	29.9	71.6	7.8*
Two diseases	2844	18.6	69.9*	8.5*
Three and more diseases	2728	17.8	66.6*	9.8*
Diseases not reported	3394	~	70.4	7.5
With functional loss (Yes)	16,030	10.9	68.9	10.2*
With functional loss (No) (Ref.)	2047	89.1	70.9	7.5
With bad memory (Yes)	2751	82.4	68.7*	8.3*
With bad memory (No) (Ref.)	13,520	17.6	75.6	5.2
Memory not reported	2352	~	76.8	9.8
No pain (=1) (Ref.)	10,545	65.2	72.0	6.4
A little pain (=2)	2915	17.6	69.2*	8.9*
Some pain (=3)	1383	8.3	65.1*	10.8*
Quite a bit pain (=4)	1051	6.0	65.1*	12.6*
A lot of pain (=5)	519	2.9	55.6*	14.4*
Pain not reported	2210	~	76.4	11.4
Excellent childhood health (=1) (Ref.)	1883	10.7	71.4	6.8
Very good childhood health (=2)	6774	38.1	72.0	7.5*
Good childhood health (=3)	4753	26.8	70.3	7.9*
Fair childhood health (=4)	3226	17.4	69.3	8.3*
Poor childhood health (=5)	1244	7.0	66.1*	9.0*
Social factors				
Married (Ref.)	15,799	84.8	70.2	7.5
Separated/divorced/widowed	2273	14.2	75.8*	9.3*
Single	155	1.0	42.0*	10.1*
No child (Ref.)	378	4.2	48.6	8.6
One child	1878	18.0	67.4*	7.3*
Two children	3616	31.6	70.8*	7.9
Three or more children	4930	46.3	73.1*	8.5
Child number not reported	7821	~	71.1	7.2
Children visiting daily or weekly (Ref.)	4652	54	74.3	8.0
Children visiting every month	1510	18.1	72.1	7.7
Children visiting every year	2236	24.9	65.0*	8.8*
Children visiting less than once per year	216	2.7	45.7*	9.3
Children visiting not reported	10,009	~	70.6	7.3
Children contacting daily or weekly (Ref.)	3568	50	70.4	7.9
Children contacting every month	2083	30.0	68.8	8.9*
Children contacting every year	421	6.1	67.2	9.9*
Children contacting less than once per year	878	14.1	70.2	8.8*
Children contacting not reported	11,673	~	71.3	7.3
With co-resident children	6144	56.9	75.4*	8.1
With children in a same neighborhood	1616	15.0	67.2*	7.8
With children in another neighborhood (Ref.)	3042	28.2	61.25	8.2
Living arrangement not reported	7830	~	71.1	7.2
Social activities leisure (Yes)	8747	47.9	71.8*	7.1*
Social activities: leisure (No) (Ref.)	9348	52.1	69.7	8.5
Social activities: helping others (Yes)	2495	13.8	71.7	7.1*
Social activities: helping others (No) (Ref.)	15,600	86.2	70.6	7.9
Social activities: learning (Yes)	999	6.3	61.1*	5.4*
Social activities: learning (No) (Ref.)	17,096	93.7	71.4	7.9

(1) *Ref*. Reference group. * indicates a significant difference from the reference group with a *p*-value< 0.10 based on the univariate regression with categorical variables. Data source: CHARLS 2013, weights are used

Next we tested stress-buffering/double jeopardy hypotheses by adding interactions between perceived future care availability and financial insecurity, functional disability, and urban/rural residence. We estimate the confounding effect of rural-urban residents, financial conditions, and ADL when they are controlled parallelly with perceived care support, while estimate the moderating effect when they are interacted with perceived care support in the model.

Since we were alert to the possibility that perceived availability of future care is endogenous to depressive symptoms--a condition that would exist if depressed individuals were less capable of mobilizing an effective support network—we also estimated regression models controlling for a lagged measure of depressive symptoms taken in 2011.

Finally, as mentioned above, we have treated respondents with missing information on family support into a “not reported” group as it is missed by random so that we can run the model with full sample size. However, robustness check has been done by assigning the reported values to other respondents within a same household. Only very small and insignificant differences were found between using the initial data and imputed data (See Table 8, 9 and 10 in Appendix 3).

## Results

### Descriptive analysis

Distributions for all study variables are shown in Table 1, as well as differences between sub-groups in average depressive symptoms and in the proportion of those who expect that future care would be available to them. Among 17,000 respondents, a large majority (70.7%) of respondents aged 45 and older expected that care would be available to them in the future.

That almost one-third of the sample did not anticipate the availability of a care provider outside of their spouses, signifies the possibility of a care-gap for older adults in Chinese society, even among those with children, particularly when their children were not living in a same city/county. Further, our univariate analysis indicated that respondents without future care expectations had significantly higher depressive scores than those who had such expectations (9.1 compared to 7.1).

We restrict our discussion of bivariate results to factors that were associated with both care expectations and depressive symptoms. Importantly, we control for these factors in regression models in order to isolate the unique contribution of care expectations in predicting depressive symptoms.

Results in Table 1 show that when compared to the youngest residents aged 45–54, those aged 55–64 were less likely to expect care availability as well as have more depressive symptoms; while those aged 75 and older were more likely to expect care availability, but had fewer depressive symptoms.. Women were more likely than men to expect future care availability but experienced more depressive symptoms. Older Chinese living in urban areas and those with at least college education were less likely to expect care availability as well as experienced less depression when compared to rural and less educated individuals, respectively.

Respondents who had two or more chronic diseases, poor/fair memory, more pain, and poor childhood health, were less likely to expect care availability and reported more depressive symptoms than their counterparts.

In terms of family factors, those who never married, were childless, and had children who visited infrequently were less likely to expect care availability and experienced more depressive symptoms when compared to their counterparts. With respect to social activities, engaging in leisure activities was associated greater likelihood of care availability and fewer depressive symptoms; in contrast engaging in educational activities was associated with less expected care availability and fewer depressive symptoms,

### Multivariate results

Estimated coefficients for perceived ability of future care (PAFC) applying various sets of control variables, both with and without controlling for lagged depressive symptoms, are shown in Table 2 (the final sample size for our final regression is *n* = 13,855 if lagged depression is not controlled, and is *n* = 10,458 if lagged depression is controlled. Estimated coefficients and standard errors for all variables are reported in Tables 4 and 5 in Appendix 1). Both contemporaneous effects of PAFC (without a lagged control) and lagged effects of PAFC (with a lagged control) are presented. The baseline model with no controls shows a significant positive relationship between PAFC and depressive symptoms. Recalling that no PAFC is coded as “1” if care was not expected, this result indicates that those who did not expect care availability had more depressive symptoms than those who expected future care support (as evidence to support Hypothesis 1). The coefficient is smaller in the lagged model compared to the contemporaneous model (1.42 to 2.09) but both are statistically significant. With age and gender (demographical variables) controlled, both contemporaneous and lagged PAFC effects increase. This suppression effect related to women having the unique combination of experiencing greater likelihood of care availability and greater depressive symptoms. Adding health variables resulted in a large reduction in the PAFC coefficients in both the contemporaneous and lagged effect models. Individually adding financial and social variables to the equation diminishes the PAFC coefficients somewhat in the contemporaneous model but not in the lagged effect model (as evidence to support Hypothesis 2).

**Table 2:** Coefficients for perceived availability of future care (PAFC) after adjusting for various control variables

Controls applied	Coefficients for PAFC predicting depression (no expected care support)	se	R-square for equation	Coefficients for PAFC predicting depression (no expected care support)	se	R-square for equation
	Lagged depression not controlled (*n* = 13,855)			Lagged depression controlled (*n* = 10,458)		
(0) None	2.09***	0.147	0.028	1.42***	0.133	0.261
(1) Predisposing	2.35***	0.104	0.063	1.48***	0.130	0.267
(2) Predisposing +Financial	2.14***	0.133	0.104	1.50***	0.128	0.283
(3) Predisposing +Health	1.65***	0.131	0.205	1.28***	0.128	0.327
(4) Predisposing +Social	2.08***	0.128	0.097	1.51***	0.127	0.281
(5) All variables	1.61***	0.120	0.244	1.30***	0.125	0.343

Itemization of control variable groups is found in Table 1, and the full estimated model results are in Tables 4 and 5 in Appendix 1. PAFC is coded as “1” if care was not expected for future need

Data source: CHARLS 2013, weights are used. Lagged depression is 2011 data

*PAFC* Perceived availability of future care, *se* standard error

* *p* < .10; *** *p* < .05; ***p < .01

When all controls are added into the final model, the PAFC coefficients are still statistically significant, though 20% lower than the bivariate model (see the bottom row of Table 2), indicating that the effect of PAFC on depression is robust to the application of a rich set of control variables as well as a lagged indicator of depressive symptoms.

We tested interaction models to examine whether financial, health, and urban/rural conditions alter the adverse impact of not expecting care on depressive symptoms. As shown in Table 3, each of the three interaction terms were tested separately in a contemporaneous model and a lagged model (the final sample size for our final regression is *n* = 13,855 if lagged depression is not controlled, and is *n* = 10,458 if lagged depression is controlled, coefficients of all variables are presented in Tables 6 and 7 in Appendix 2). In the contemporaneous model (first two columns of numbers in Table 3), all interactions tested were statistically significant and in a direction that suggests the impact of lacking an expected care provider on depressive symptoms was more severe for those with functional impairment, worse financial status, and rural residency (as evidence to support Hypothesis 3). In models with lagged depressive symptoms controlled, only functional impairment significantly interacted with PAFC.

**Table 3:** Coefficients for interactions between perceived availability future care (PAFC) and resource/deficit variables

Interaction terms	Interactions predicting depressive symptoms			
	Lagged depression not controlled		lagged depression controlled	
	(*n* = 13,855)		(*n* = 10,458)	
	b	se	b	se
No expected care & rural residence	.487*	0.245	0.032	0.251
(PAFC interacted by rural/urban residence)				
No expected care & worse living standard (PAFC interacted by financial strain)	.732**	0.311	0.679	0.462
No expected care & ADL (PAFC interacted by limitation in activities of daily living)	1.503**	0.597	1.255**	0.618

Itemization of control variable groups is found in Table 1, and the full estimated model results are in Tables 6 and 7 in Appendix 2; Each of the interaction terms was controlled separately into the final model. PAFC is coded as “1” if care was not expected for future need

Data source: CHARLS 2013, weights are used. Lagged depression is 2011 data

PAFC Perceived availability of future care, b estimated coefficient, se standard error, ADL Limitation in daily living activities

* *p* < .10; *** *p* < .05; ***p < .01

**Fig. 1:**
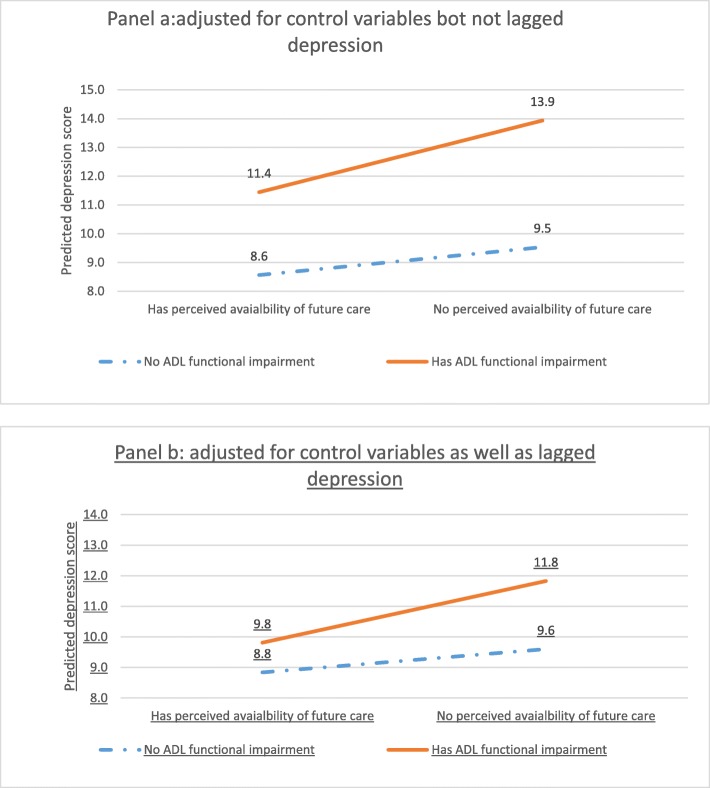
Predicted depressive score for interaction group by perceived availability of future care and functional impairment (Panel **a**: adjusted for control variables but not lagged depression) and (Panel **b**: adjusted for control variables as well as lagged depression). Notes: Predicted depression score was calculated based on the significant estimated coefficients Tables 6 and 7 in Appendix 2 and mean values of all the significant predictors. Data source: CHARLS 2013, weights are used

Based on estimated coefficients from the full model, we calculated and then plot predicted depressive symptom scores formed by the interaction between ADL functional impairment and PAFC, holding all covariates at their mean values. These predicted values are presented in panel A of Fig. 1 without controlling for lagged depression (based on coefficients shown in Table 6 in Appendix 2) and panel B of Fig. 1 with a control for lagged depression (based on coefficients shown in Table 7 in Appendix 2). These figures reveal a pattern characteristic of buffering or double jeopardy, although somewhat weaker in strength with lagged depression controlled. Among those respondents who were functionally healthy, there is little change in depressive symptoms by whether or not future care was expected. However, in the presence of disability, not anticipating a care provider was particularly disadvantageous. Viewed another way, the combination of disability and the absence of a future care provider produced elevated depressive symptoms, a form of double jeopardy in the consequences of expecting unmet need.

## Discussion

This investigation examined depressive symptomology among middle-aged and older adults in China as related to their expectation of having a care provider available to them. We found a strikingly large percentage—almost one-third, of the Chinese population aged 45 and older—that did not expect to have a care provider in the event of future need. Even with the rapidity of social, economic, and family change in China, such a high prevalence of uncertainty is surprising in a nation still guided by the precepts of filial piety, where children are expected to provide for their parents’ needs.

Most notable among our findings was the persistence of care expectations in predicting depressive symptoms, even after controlling for a large range of economic, health, and social factors as well as a lagged predictor of the outcome. Although about one third of the effect of perceived availability of future care on depressive symptoms was explained by these covariates, the robustness of this findings provides evidence for the utility of social support and control theories as explanatory paradigms. We infer from our results that uncertainty about having a care provider to meet basic needs in later life weakens one’s sense that the future is predictable and controllable, thereby inducing distress. That the expectation of a future care deficit is tied to a demonstrable outcome confirms the power of subjective appraisals to influence affective states, harkening back to the observation of Thomas and Thomas [42] that “if (individuals) define situations as real, they are real in their consequences” (p. 572).

Our findings also indicate that elevated emotional distress among those with uncertainty about having a future care provider is partially explained by the absence of children and, among those with children, having infrequent contact with them and living farther from them. Given declining fertility rates in China, these findings suggest that the anticipation of unmet need may surge in the older population with concomitant consequences for their mental health.

Our test of resource-buffering and double-jeopardy hypotheses was most strongly observed with respect to functional health. Functionally healthy individuals have the physical resources to resist or delay consideration of the consequences of lacking a future care provider. Alternatively, functionally impaired individuals are prone to realizing the true implications of being absent a caregiver. Interactions with financial security and urban/rural residence were fully explained by the lagged measure of depressive symptoms. That is, pre-existing depression in the high-risk groups formed by the intersection of care uncertainty with financial stress and rural residency provided an alternative explanation for data patterns, affirming the utility of a longitudinal approach with lagged predictors.

Our findings that ADL and worse living standard could increase depression generated by the lack of PFCS is consistent to the findings in Bangerter et al. [12] that bad physical health and low material support could increase depression of children generated by mother’s problems. However, the moderating effect of urban /rural residence on depression found in our study is new and unique in literature.

Several limitations of our investigation deserve mention. First, our measure of perceived future care availability was limited to one question with a dichotomous response option. Thus, it was not possible to discern the *degree* of uncertainty or assess the reliability of this indicator as a true measure of uncertainty.

Second, we did not assess whether the expectation of a care deficit prospectively results in an actual care deficit, leaving open the possibility that individuals without an expectation for care will eventually recruit a care provider from their informal network. Alternatively, those individuals without such an expectation may have their needs met by formal care services. Further, some individuals who expect to have a care provider may be disappointed and not have that individual available when needed.

Finally, future cohorts of older adults in China will meet the needs of old age care under very different conditions than existed for the current cohort, having been exposed to a relatively more prosperous economy, having smaller families, and experiencing new forms of filial piety. These historical exposures may produce new challenges, but also new opportunities for older adults in meeting the needs of later life. Economic growth will deliver financial resources to older adults that may mitigate some of the disadvantages a declining supply of offspring. In addition, the Chinese government is beginning to develop home- and community-based services as well as a residential old age care system that, while still limited, are likely to be further developed to the benefit older adults in the future.

## Conclusions

Our major findings suggest that uncertainty about future care pervades a large segment of the older population of China, and is not without consequences for emotional well-being. Given the aging of the Chinese population, the attendant growth in the chronic disease burden [43], and attenuation of filial resources, the government will likely develop policies to meet the expected growth in unmet need. Such policy initiatives include financially incentivizing adult children to continue their caregiving roles, developing community-based care, instituting a long-term care insurance program, and providing low-cost service-enriched housing for frail older people who do not have sufficient family support. In addition, our results underscore the importance for China of developing mental health services for middle-aged and older individuals, particularly targeting older adults with health difficulties and limited family support.

To the degree that uncertainty with regard to future care adversely influences mental health, we suggest that greater attention be devoted to addressing the implications of weak formal and informal safety nets for older adults. Meeting mental health needs in later life may become more challenging as the pace of economic development, cultural change, and urbanization accelerates in China. We suggest that future research explore how expectations for care have shifted in relation to these new contingencies, and their implications for the psychological and physical well-being of older adults in China, as well as in other middle-income and emerging economies undergoing similar demographic and social change.

## Data Availability

The CHARLS survey data used in this study are confidential and cannot be shared. Researchers need to apply for and obtain approval to access and use this survey data for research purpose from the CHARLS team at the Beijing University. More detailed information on CHARLS data is provided on http://charls.pku.edu.cn/index.html. However, the detailed results from the full regression models in the current study are available as additional supporting tables or on a reasonable request to the corresponding author at cathy.gong@anu.edu.au.

## Appendix 1

**Table 4:** Joint predictors of depression score among older Chinese (with all control variables)

DV: Depression score	Model 1		Model 2		Model 3		Model 4		Model 5	
	b	se	b	se	b	se	b	se	b	se
(0) Depression score at baseline	No		No		No		No		No	
(1) Expected future support for ADL care needs (no)	2.353***	0.102	2.135***	0.133	1.645***	0.131	2.076***	0.128	1.610***	0.121
(2) Predisposing factors	Yes	Yes	Yes	Yes			Yes	Yes		
Aged 45–54										
Aged 55–64	0.675***	0.112	0.465***	0.143	0.086	0.136	0.534***	0.137	−0.120	0.134
Aged 65–74	0.996***	0.132	0.710***	0.167	−0.297*	0.157	0.523***	0.171	−0.615***	0.159
Aged 75+	0.504***	0.186	0.148***	0.229	−1.237***	0.217	−0.468*	0.242	−1.941***	0.229
Male										
Female	1.848***	0.094	1.478***	0.120	1.024***	0.115	1.677***	0.113	0.853***	0.115
(3) Financial factors	No		Yes		No		No		Yes	
Rural										
Urban			−1.138***	0.127					−0.730***	0.116
Under primary schooling										
Second schooling			−1.131***	0.131					−0.582***	0.128
College and above degree			−2.143***	0.301					−0.516	0.328
Better living standard										
About same living standard			0.237	0.268					0.143	0.252
Worse living standard			1.994***	0.260					1.282***	0.244
(4) Health factors	No		No		Yes		No		Yes	
With ADLs					1.608***	0.307			1.624***	0.302
With IADLs					2.373***	0.186			2.049***	0.186
No disease										
One disease					0.608***	0.142			0.616***	0.137
Two diseases					0.943***	0.174			0.941***	0.164
Three and more diseases					1.590***	0.196			1.664***	0.180
With functional loss					1.381***	0.179			1.305***	0.175
With bad memory					1.782***	0.125			1.593***	0.120
Level of pain (1–5)					0.166***	0.042			0.147***	0.039
Childhood health (1 = excellent, 5 = poor)					0.252***	0.050			0.196***	0.048
(5) Social factors	No		No		No		Yes		Yes	
Married (omitted)										
Separated/divorced/widowed							1.301***	0.218	1.129***	0.196
Single							2.414**	0.877	1.180	0.828
No child (omitted)										
One child							0.022	0.519	0.253	0.499
Two children							0.407	0.524	0.408	0.503
Three or more children							0.780	0.547	0.606	0.528
Children visiting daily/weekly (omitted)										
Children visiting monthly							−0.267	0.269	−0.193	0.239
Children visiting every year							0.611**	0.248	0.513**	0.212
Children visiting less than once per year							0.746	1.001	0.903	0.908
Children contacting daily/weekly (omitted)										
Children contacting monthly							0.623*	0.318	0.462**	0.198
Children contacting every year							1.246***	0.480	0.866**	0.375
Children contacting less than once per year							0.312	0.437	0.040	0.338
Children: co-residing in same household							−0.069	0.191	−0.235	0.181
Children living in a same neighbourhood							−0.638**	0.287	−0.495**	0.248
Social activities: leisure							−0.112**	0.116	−0.716***	0.111
Social activities: helping others							−0.126	0.152	0.100	0.145
Social activities: learning							−1.977***	0.279	−0.799***	0.285
Cons	7.970	0.121	7.828	0.314	5.686	0.265	8.261	0.625	5.907	0.616
Sample size	14,179		13,896		13,855		13,896		13,855	
R-Square	0.0619		0.1042		0.205		0.107		0.244	

(1) * indicate statistically significant at 10%, ** indicate significant at 5%, and *** indicate significant at 1%. (2) Model 1 controls for expected future care support and predisposing variables. Based on Model 1, Model 2 adds financial variables; Model 3 adds health variables; Model 4 adds social variables; Model 5 adds all financial, health and social factors. (3) Reference groups in each category are: Aged 45–54, male, rural, under primary schooling, better living standard, without ADLs, without IADLs, without disease, without functional loss, without bad memory, excellent childhood health, married with spouse presented, without child, children visiting daily or weekly, children contacting daily or weekly, no social activities for leisure, no social activities for helping others, no social activities for learning. (4) The “not reported” group in each category is treated as a separated group in order to maintain a full sample size for estimation. (5) Lagged depression scores are not controlled for in Models 1–5. Data source: CHARLS 2013, weights are used

**Table 5:** Joint predictors of depression score among older Chinese (with all control variables and lagged depression)

DV: Depression score	Model 6		Model 7		Model 8		Model 9		Model 10	
	b	se	b	se	b	se	b	se	b	se
(0) Depression score at baseline	0.436***	0.010	0.410***	0.010	0.360***	0.011	0.420***	0.010	0.333***	0.011
(1) Expected future support for ADL care needs (no)	1.481***	0.130	1.503***	0.128	1.279***	0.128	1.508***	0.127	1.302***	0.125
(2) Predisposing factors										
Aged 45–54 (Ref.)										
Aged 55–64	0.467***	0.143	0.325**	0.148	0.126	0.143	0.400***	0.139	0.053	0.141
Aged 55–74	0.487***	0.164	0.345**	0.169	−0.233	0.164	0.298	0.183	−0.358**	0.177
Aged 75+	0.162	0.237	−0.040	0.241	−0.881***	0.233	−0.266	0.259	−1.190***	0.251
Male (Ref.)										
Female	0.963***	0.113	0.842***	0.115	0.616***	0.111	0.924***	0.111	0.538 ***	0.113
(3) Financial factors										
Rural (Ref.)										
Urban			−0.595***	0.131					− 0.515 ***	0.124
Under primary schooling (Ref.)										
Second schooling			−0.517***	0.133					−0.318**	0.135
College and above degree			−0.897***	0.314					−0.088	0.351
Better living standard (Ref.)										
About same living standard			0.370	0.264					0.292	0.258
Worse living standard			1.436***	0.260					1.051***	0.255
Not reported			1.165***	0.340					0.694**	0.318
(3) Health factors										
With ADLs					1.198***	0.319			1.191***	0.317
With IADLs					1.543***	0.202			1.423 ***	0.201
No disease (Ref.)										
One disease					0.358**	0.140			0.401 ***	0.137
Two diseases					0.375**	0.167			0.434 ***	0.159
Three and more diseases					0.495***	0.183			0.652 ***	0.175
Diseases not reported					0.111	0.917			0.187	0.935
With functional loss					1.082***	0.189			1.051 ***	0.187
With bad memory					1.371***	0.130			1.289 ***	0.128
Level of pain (0–5)					0.135***	0.037			0.127 ***	0.035
Childhood health (from excellent to poor)					0.041	0.052			0.021	0.049
(4) Social factors										
Married (Ref.)										
Separated/divorced/widowed							0.651***	0.214	0.680 ***	0.206
Single							0.812	0.963	0.293	0.923
No child (Ref.)										
One child							0.485	0.694	0.516	0.640
Two children							0.786	0.689	0.751	0.635
Three or more children							1.043	0.698	0.903	0.648
Not reported							3.703	3.485	4.763	3.390
Children visiting daily or weekly (Ref.)										
Children visiting every month							−0.148	0.247	− 0.099	0.242
Children visiting every year							0.352	0.234	0.280	0.223
Children visiting less than once per year							−0.041	0.856	0.082	0.802
Not reported							0.495	0.274	0.584	0.264
Children contacting daily or weekly (Ref.)										
Children contacting every month							0.268	0.204	0.248	0.197
Children contacting every year							0.407	0.414	0.364	0.386
Children contacting less than once per year							−0.239	0.436	−0.379	0.416
Not reported							0.025	0.240	0.221	0.230
Children living in a other neighbourhood/city/county (Ref.)										
Children co-resident							0.063	0.184	−0.143	0.180
Children living in a same neighbourhood							−0.479*	0.252	−0.435 *	0.244
Social activities: leisure							− 0.682***	0.120	−0.509***	0.114
Social activities: helping others							− 0.073	0.155	0.138	0.148
Social activities: learning							− 1033***	0.283	−0.389	0.290
Cons	4.454	0.166	4.293	0.319	3.895	0.248	4.296	0.718	3.429	0.734
Sample size	10,458		10,458		10,458		10,458		10,485	
R-Square	0.27		0.283		0.327		0.281		0.343	

(1) * indicate significant at 10%, ** indicate significant at 5%, and *** indicate significant at 1%. (2) Model 6 controls for lagged depression score, expected future care support and predisposing variables. Based on Model 6, Model 7 adds financial variables only; Model 8 adds health variables only; Model 9 adds social variables only; Model 10 adds all financial, health and social factors. (3) Reference groups in each category: Aged 45–54, male, rural, under primary schooling, better living standard, without ADLs, without IADLs, without disease, without functional loss, without bad memory, excellent childhood health, socially married, without child, children visiting daily or weekly, children contacting daily or weekly, no social activities: leisure, no social activities: helping others, no social activities: learning. (4) The not reported group in each category is treated as a separated group in the model in order to maintain a full sample size for estimation. Data source: CHARLS 2013, weights are used. Lagged depression scores are from 2011

## Appendix 2

**Table 6:** Joint predictors of depression score among older Chinese (with all control variables and interactions)

DV: depression score	Model 11		Model 12		Model 13	
	b	se	b	se	b	se
(0) Depression score at baseline	No		No		No	
(1.1) Expected future care support (no)	1.308***	0.271	1.331***	0.200	0.975***	0.191
(1.2) Interaction items						
With support & better living standard (Ref.)						
With support & average living standard	0.345	0.380				
With support & worse living standard						
No support & better living standard	−0.700	0.590				
No support & average living standard						
No support & worse living standard	0.732**	0.311				
With support & urban						
With support & rural						
No support & urban						
No support & rural			0.487*	0.245		
With support & no ADL						
With support & with ADL						
No support & no ADL					0.871***	0.247
No support & with ADL						
(2) Predisposing factors					1.503**	0.597
Aged 45–54						
Aged 55–64	−0.139	0.132	−0.124	0.133	−0.121	0.133
Aged 55–74	− 0.642***	0.156	−0.618***	0.159	−0.625***	0.159
Aged 75+	−1.963***	0.228	−1.952***	0.227	−1.948***	0.228
Male						
Female	0.852***	0.113	0.849***	0.114	0.855***	0.114
Rural						
Urban	−0.719***	0.114	−1.068***	0.216	−0.717***	0.115
(3) Financial factors						
Under primary schooling						
Second schooling	−0.590***	0.126	−0.580***	0.128	−0.588***	0.128
College and above degree	−0.530*	0.320	−0.504	0.326	−0.512	0.327
Better living standard						
About same living standard	−0.325	0.445	0.142	0.251	0.144	0.253
Worse living standard	1.580***	0.393	1.278***	0.243	1.274***	0.244
(3) Health factors						
Without ADLs						
With ADLs	1.627***	0.300	1.607***	0.302	2.872***	0.502
ADLs not reported	−2.157***	0.118	−2.160***	0.119		
With IADLs	2.035***	0.185	2.049***	0.186	2.042***	0.186
No disease						
One disease	0.618***	0.136	0.616***	0.137	0.614***	0.136
Two diseases	0.942***	0.161	0.938***	0.164	0.957***	0.162
Three and more diseases	1.655***	0.177	1.662***	0.180	1.658***	0.180
With functional loss	1.307***	0.175	1.307***	0.175	1.032***	0.175
With bad memory	1.602***	0.119	1.598***	0.119	1.064***	0.119
Level of pain (0–5)	0.146***	0.039	0.147***	0.039	0.145***	0.039
Childhood health (1 = excellent, 5 = poor)	0.198***	0.047	0.196***	0.047	0.194***	0.047
(4) Social factors						
Married (omitted)						
Separated/divorced/widowed	1.142***	0.194	1.142***	0.195	1.149***	0.194
Single	1.154	0.823	1.116	0.830	1.205	0.836
No child (omitted)						
One child	0.223	0.503	0.226	0.505	0.218	0.518
Two children	0.362	0.509	0.374	0.511	0.356	0.522
Three or more children	0.538	0.532	0.560	0.534	0.546	0.544
Children visiting daily or weekly (omitted)						
Children visiting every month	−0.205	0.236	−0.200	0.239	−0.194	0.238
Children visiting every year	0.518**	0.212	0.520**	0.213	0.538**	0.213
Children visiting less than once per year	0.942	0.845	0.986	0.893	0.979	0.854
Children contacting daily or weekly (omitted)						
Children contacting every month	0.461**	0.196	0.466 **	0.199	0.461**	0.197
Children contacting every year	0.873**	0.375	0.874 **	0.376	0.894**	0.375
Children contacting less than once per year	0.037	0.329	0.050	0.336	0.056	0.333
Children co-residing in same household	−0.209	0.176	−0.233	0.180	−0.214	0.179
Children living in a same neighbourhood	−0.466*	0.241	−0.496*	0.247	−0.485**	0.242
Social activities for leisure	−0.716***	0.110	−0.710***	0.110	−0.716***	0.111
Social activities for helping others	0.078	0.142	0.097	0.143	0.086	0.144
Social activities for learning	−0.757***	0.271	−0.778***	0.278	−0.770***	0.284
Cons	5.866		6.000		5.144	
Sample size	13,855		13,855		13,855	
R-Square	0.2459		0.2447		0.2456	

(1) * indicate statistically significant at 10%, ** indicate significant at 5%, and *** indicate significant at 1%. (2) Based on Model 5, Model 11 further controls for the interaction term of expected future care support with perceived living standard; Model 12 controls for interaction term with urban/rural residency; Model 13 controls for interaction term with ADL. (3) Reference groups in each category are: Aged 45–54, male, rural, under primary schooling, better living standard, without ADLs, without IADLs, without disease, without functional loss, without bad memory, excellent childhood health, married with spouse presented, without child, children visiting daily or weekly, children contacting daily or weekly, no social activities for leisure, no social activities for helping others, no social activities for learning. (4) The “not reported” group in each variable is treated as a separated group in order to maintain full sample size for estimation. (5) Lagged depression scores are not controlled for in Models 11–13.Data source: CHARLS 2013, weights are used. Lagged depression scores are from 2011

**Table 7:** Joint predictors of depression score among older Chinese (with all controls, interactions, lagged depression)

DV: depression score	Model 21		Model 22		Model 23	
	b	se	b	se	b	se
Depression score-baseline	0.332***	0.011	0.333***	0.011	0.332***	0.011
(0) Expected future care support (no)	0.881**	0.440	1.285***	0.202	0.762***	0.188
(1) Interaction items						
With support & better living standard (Ref.)						
With support & average living standard	−0.010	0.494				
With support & worse living standard						
No support & better living standard	−0.399	0.696				
No support & average living standard						
No support & worse living standard	0.679	0.462				
With support & urban (Ref.)						
With support & rural						
No support & urban						
No support & rural			0.032	0.251		
With support & no ADL (Ref.)						
With support & with ADL						
No support & no ADL					0.720***	0.244
No support & with ADL					1.255**	0.618
(2) Predisposing factors						
Aged 45–54						
Aged 55–64	0.038	0.139	0.047	0.140	0.051	0.140
Aged 55–74	−0.382**	0.174	−0.367**	0.176	−0.370**	0.176
Aged 75+	−1.213***	0.249	−1.204***	0.250	−1.209***	0.249
Male						
Female	0.537***	0.113	0.536***	0.113	0.538***	0.113
Rural						
Urban	−0.509***	0.122	−0.532**	0.215	−0.504***	0.123
(3) Financial factors						
Under primary schooling						
Second schooling	−0.324**	0.133	−0.318**	0.135	−0.325**	0.135
College and above degree	−0.088	0.347	−0.078	0.355	−0.078	0.350
Better living standard						
About same living standard	0.167	0.557	0.294	0.258	0.295	0.258
Worse living standard	1.400***	0.534	1.050***	0.255	1.052***	0.255
Not reported	0.000	(omitted)	0.703**	0.318	0.704**	0.318
(3) Health factors						
Without ADLs						
With ADLs	1.194***	0.316	1.189***	0.317	0.972**	0.395
ADLs not reported	−1.483***	0.115	−1.480***	0.116	0.000	(omitted)
With IADLs	1.413***	0.201	1.422***	0.201	1.416***	0.201
No disease						
One disease	0.403***	0.136	0.401***	0.137	0.399***	0.136
Two diseases	0.437***	0.158	0.434***	0.159	0.448***	0.158
Three and more diseases	0.649***	0.173	0.651***	0.175	0.653***	0.175
Diseases not reported	0.173	0.932	0.174	0.939	0.132	0.941
With functional loss	1.051***	0.187	1.049***	0.187	1.044***	0.186
With bad memory	1.300***	0.127	1.288***	0.128	1.295***	0.127
Level of pain (0–5)	0.126***	0.035	0.127***	0.035	0.125***	0.035
Childhood health (1 = excellent, 5 = poor)	0.022	0.049	0.021	0.049	0.020	0.049
(4) Social factors						
Married (omitted)						
Separated/divorced/widowed	0.691 ***	0.204	0.685***	0.205	0.691***	0.204
Single	0.288	0.921	0.289	0.924	0.344	0.924
No child (omitted)						
One child	0.500	0.641	0.502	0.641	0.524	0.642
Two children	0.715	0.638	0.724	0.636	0.740	0.639
Three or more children	0.848	0.652	0.865	0.650	0.882	0.652
Not reported	2.856	4.464	3.092	4.519	3.283	4.495
Children visiting daily or weekly (omitted)						
Children visiting every month	−0.098	0.242	−0.096	0.244	−0.093	0.243
Children visiting every year	0.296	0.223	0.296	0.224	0.304	0.223
Children visiting less than once per year	0.177	0.766	0.143	0.796	0.208	0.760
Not reported	0.654***	0.282	0.658**	0.283	0.642**	0.282
Children contacting daily or weekly (omitted)						
Children contacting every month	0.256	0.196	0.255	0.198	0.254	0.196
Children contacting every year	0.386	0.387	0.384	0.388	0.394	0.387
Children contacting less than once per year	−0.360	0.399	−0.348	0.404	−0.358	0.401
Not reported	0.242	0.236	0.249	0.238	0.262	0.238
Children co-residing in same household	−0.122	0.179	−0.131	0.181	−0.131	0.180
Children living in a same neighbourhood	−0.417*	0.240	−0.430*	0.244	−0.431*	0.240
Social activities: leisure	−0.511***	0.114	−0.512***	0.114	−0.513***	0.114
Social activities: helping others	0.125	0.147	0.130	0.147	0.130	0.146
Social activities: learning	−0.366	0.280	−0.377	0.288	−0.367	0.287
Cons	3.172	0.847	3.345	0.749	2.725	0.761
Sample size	10,458		10,458		10,458	
R-Square	0.344		0.344		0.344	

(1) * indicate statistically significant at 10%, ** indicate significant at 5%, and *** indicate significant at 1%. (2) Based on Model 5, Model 11 further controls for the lagged depression, interaction term of expected future care support with perceived living standard; Model 12 controls for interaction term with urban/rural residency; Model 13 controls for interaction term with ADL. (3) Reference groups in each category are: Aged 45–54, male, rural, under primary schooling, better living standard, without ADLs, without IADLs, without disease, without functional loss, without bad memory, excellent childhood health, married with spouse presented, without child, children visiting daily or weekly, children contacting daily or weekly, no social activities for leisure, no social activities for helping others, no social activities for learning. (4) The “not reported” group in each variable is treated as a separated group in order to maintain full sample size for estimation. Data source: CHARLS 2013, weights are used. Lagged depression scores are from 2011

## Appendix 3

**Table 8:** Perceived availability of future care and mean depression scores by imputed family support characteristics

Imputed family support data Sample characteristics	Sample size (n)	Population distribution (%)	Perceived future care availability (%)	Depression score (0–30)
No child (Ref.)	504	3.2	52.1	8.2
One child	3301	18.3	66.5*	6.9*
Two children	6533	33.4	72.2*	7.6
Three or more children	8264	45.0	72.4*	8.2
Children visiting daily (Ref.)	5261	36.2	74.3	7.7
Children visiting weekly	2626	17.1	73.1	7.4
Children visiting every month	2625	18.4	72.2	7.6
Children visiting every year	3970	25.8	66.2*	8.5*
Children visiting less than once per year	357	2.6	46.0*	9.4
Children contacting every day (Ref.)	1073	8.6	75.1	7.7
Children contacting every week	5255	43.5	70.6	7.7
Children contacting every month	3631	30.4	68.0	8.6*
Children contacting every year	671	5.6	66.8	9.5*
Children contacting less than once per year	1305	11.9	69.0	8.4*
With co-resident children (Ref.)	10,597	58.3	74.9*	7.7
With children living in a same neighbourhood	2750	14.0	67.9*	7.6
With children living in other neighbourhood/city/county (Ref.)	5255	27.7	63.1	7.9

Data source: CHARLS 2013 with imputed family support data for missing values, weights are used

(1) *Ref*. reference group. * indicates a significant difference from the reference group with a *p*-value< 0.10 based on the univariate regression with categorical variables

**Table 9:** Coefficients for perceived availability of future care (PAFC) after adjusting for various control variables

Controls applied	Coefficients for PAFC predicting depression (no expected care support)	se	R-square for equation	Coefficients for PAFC predicting depression (no expected care support)	se	R-square for equation
	Lagged depression not controlled			Lagged depression controlled		
	(*n* = 13,855)			(*n* = 10,458)		
(0) None	2.085***	0.147	0.028	1.424***	0.133	0.261
(1) Predisposing	2.182***	0.136	0.077	1.516***	0.129	0.274
(2) Predisposing +financial	2.135***	0.133	0.104	1.503***	0.128	0.283
(3) Predisposing +health	1.692***	0.128	0.215	1.312***	0.126	0.330
(4) Predisposing +social	2.106***	0.129	0.129	1.532***	0.128	0.283
(5) All variables	1.620***	0.122	0.243	1.302***	0.125	0.342

Itemization of control variable groups is found in Table 1, and the full estimated model results are in Tables 4 and 5 in Appendix 1. PAFC = 1 if no care support. Data source: CHARLS 2013 with imputed family support data, weights are used. Lagged depression is derived from 2011 data

*PAFC* Perceived availability of future care, *se* standard error

* *p* < .10; *** *p* < .05; ****p* < .01

**Table 10:** Coefficients for interactions between perceived availability future care (PAFC) and resource/deficit variables

Interaction terms	Interactions predicting depressive symptoms			
	Lagged depression not controlled		Lagged depression controlled	
	(*n* = 13,855)		(*n* = 10,458)	
	b	se	b	se
No care support & rural residence (PAFC interacted by rural/urban residence)	.483**	0.246	0.031	0.251
No care support &worse living standard (PAFC interacted by financial strain)	.731**	0.312	0.722**	0.29
No care support & with ADL (PAFC interacted by limitation in activities of daily living)	1.497**	0.596	1.255**	0.618

Itemization of control variable groups is found in Table 1, and the full estimated model results are in Appendix 2.1 and 2.2; Each of the interaction terms was controlled separately into the final model. PAFC = 1 if no care support. Data source: CHARLS 2013 with imputed family support data, weights are used. Lagged depression from 2011

*PAFC* Perceived availability of future care, *b* estimated coefficient, *se* standard error

* *p* < .10; *** *p* < .05; ****p* < .01

## Appendix 4

**Table 11:** Significant factors influencing depression symptoms among older people in Asian countries (China, Korea and Viet) from previous studies

		China			Korea	Viet
Literature		Zurlo et al., 2014	Shen 2014	Lei et al. 2014	Lee and Smith 2011	Leggett, 2012
Data and measurement	Data	CHARLS 2011	CHARLS 2008 pilot survey	CHARLS 2011	KLSoA 2006	A representative sample of 600
	Population	Aged 45+	Aged 45+	Aged 45+	Aged 45+	Aged 55+
	ID = Depression score	CES-D10	CES-D7	CES-D10	CES-D10	CES-D20
Predisposing factors	Age	*				
	Gender	*	*	*		*
	Urban/rural	*				
	Province		*			
Socio-economic status	Education	*	*	*	*	*
	Material hardship/expenditure		*	*	*	*
	Subsidy for aged 60+ (rural)	*				
	Age pension (urban)	*				
Physical health	Childhood health			*		
	Physical health					
	Chronic diseases	*	*	*	*	*
	ADLs	*		*		*
	Pains			*		*
	Disability/functional problem			*		
Social support	Perceived future care support		*			
	Emotional support (feel loved and cared for)					*
	Marital status (living with a spouse)	*	*			
	Lack of contact of children	*				
	# of extended family and relative (rural)	*				
	Activity Participation last week	*				
	Senior centre in the community (Rural)	*				
	Number of community amenities		*			

*CES-D* indicates the Centre for Epidemiologic Studies Depression Scale

*indicates significant correlations

## Abbreviations

ADL: Limitations in activities of daily living
CHARLS: China Health and Retirement Longitudinal Study
IADL: Limitations in instrumental activities of daily living
OLS: Ordinary Least Squares
PAFC: Perceived availability of future care
SE: Standard Error
SES: Socio-economic status

## Footnotes

1 Care uncertainty is a risk factor to emotional distress as according to Chinese traditional culture, care is expected mostly from children when parents become old or disabled. Distress is usually associated with one or series of negative events and will usually go away with change in environment or removal of stressor. Professional intervention and medications are not usually necessary, but actual and perceived social supports such as usual friendship and family networks help. Depression could be the result of persistent and long-term emotional distress.

2 In this study, instead of using different variables (pension, social insurance, and medical insurance) to measure financial status, we use one variable “relative living standard”, a subjective measure of financial status which takes into account of different income resources, housing, savings, and living costs etc. Using one comprehensive variable can help us to simplify the measure of financial status and to estimate it’s the moderating effects using the interaction terms.
